# The association of HDL-apoCIII with coronary heart disease and the effect of statin treatment on it

**DOI:** 10.1186/s12944-015-0129-8

**Published:** 2015-10-09

**Authors:** Xiaowei Xiong, Hong Liu, Lu Hua, Hui Zhao, Dongxue Wang, Yishi Li

**Affiliations:** The Key Laboratory of Clinical Trial Research of Cardiovascular Drugs, Ministry of Health, State Key Laboratory of Cardiovascular Diseases, Fuwai Hospital, National Center for Cardiovascular Diseases, Chinese Academy of Medical Sciences and Peking Union Medical College, Beijing, China; Department of Cardiology, Wuxi People’ Hospital Affiliated to Nanjing Medical University, Wuxi, Jiangsu China

**Keywords:** Coronary heart disease (CHD), Apolipoprotein CIII (apoCIII), Statin, High density lipoprotein (HDL)

## Abstract

**Background:**

Apolipoprotein CIII (apoCIII) is considered to impair the anti-atherogenic effect of high density lipoprotein (HDL) in coronary heart disease (CHD) patients, and apoCIII content in HDL (HDL-apoCIII) predicts CHD more accurately. However, the relationship between HDL-apoCIII and CHD, and the effect of statin treatment on HDL-apoCIII are still unclear. The aims of the study are to establish the association of HDL-apoCIII with CHD, and investigate the effect of statin treatment on HDL-apoCIII in CHD patients.

**Methods:**

We conducted a hospital-based observational study. Totally 80 non-CHD patients and 120 CHD patients without statin treatment were previously enrolled in this study. All the CHD patients received statin treatment, and 63 of them were followed after 3 months of regular statin treatment. HDL sample of each patient was isolated by density gradient ultracentrifugation from fasting venous plasma, and HDL-apoCIII of each patient was measured by ELISA method.

**Results:**

HDL-apoCIII was significantly higher in CHD patients than non-CHD patients (*p* < 0.05), and it was still an independent predictor of CHD after adjusting for other factors. Total plasma apoCIII, especially HDL-apoCIII was significantly elevated after statin treatment in CHD patients, whereas total cholesterol (TC), low density lipoprotein cholesterol (LDL-c) and apolipoprotein B (apoB) were decreased significantly (*p* < 0.05). Compared with CHD patients without diabetes mellitus (DM), the effect of statin treatment on apoCIII markers was minor in CHD patients with DM. And HDL-apoCIII correlated with plasma TG significantly in non-CHD and CHD patients (*p* < 0.05), but the correlation in CHD patients did not exist after statin treatment (*p* > 0.05).

**Conclusions:**

HDL-apoCIII has a significant and positive association with CHD. Although conventional atherogenic lipid markers have a significantly decrease in CHD patients after statin treatment, HDL-apoCIII has a further elevation at the same time.

**Electronic supplementary material:**

The online version of this article (doi:10.1186/s12944-015-0129-8) contains supplementary material, which is available to authorized users.

## Introduction

Coronary heart disease (CHD) is a chronic inflammatory disease characterized with dyslipidemia [1–3]. Conventional lipid markers such as total cholesterol (TC), triglyceride (TG), low-density lipoprotein cholesterol (LDL-c) and high-density lipoprotein cholesterol (HDL-c) are considered to associate with CHD positively or inversely [4, 5]. However, not all the CHD patients have dyslipidemia, and large clinical trials also fail to find more heart benefits by further elevating HDL-c [6, 7]. Increasing evidences suggest that apolipoproteins in lipoproteins or plasma may predict CHD better [5, 8], and the biological function of HDL is associated with its components much more [9].

As a major component of triglyceride-rich lipoproteins (TRL), apolipoprotein CIII (apoCIII) is considered to participate in the process of CHD by disturbing lipids lipolysis and promoting inflammation response [10–12]. Studies found that apoCIII or apoCIII-containing lipoproteins had significant associations with CHD [8, 13, 14]. Even the antiatherogenic function of HDL was also impaired by apoCIII in CHD patients [15], and elevated apoCIII content in HDL (HDL-apoCIII) predicted the occurrence and progression of CHD in prospective or retrospective studies [16–18]. However, the relationship between HDL-apoCIII and CHD is still inconclusive because of the general usage of lipid-lowering drugs in almost all studies above. Lipid-lowering drugs, particularly statins were demonstrated to reduce the progression of CHD and cardiovascular event significantly by decreasing atherogenic lipids [19, 20]. To date, it is still unclear if statin treatment has the same effect on HDL-apoCIII in CHD patients. The objectives of our study are to establish the association between HDL-apoCIII and CHD, and investigate the effect of statin treatment on HDL-apoCIII in CHD patients.

## Method

### Study design and population

The study was approved by the Ethics Committee of the Fuwai Hospital (Approval No. 2012–382) and was registered at http://clinicaltrials.gov/ (Identification Number: NCT 01543308). A written informed consent was provided to all study patients, and the study protocol conforms to the guidelines of Declaration of Helsinki.

We conducted a hospital-based observational study. Patients without previously lipid-lowering treatment were consecutively recruited from April 2012 to March 2013 in Ward IV of Fuwai Hospital. Total 200 patients, including 80 non-CHD patients and 120 CHD patients, were entered into the study. CHD was diagnosed according to the angiographic results: stenosis ≥ 50 % in at least one major coronary artery. All 120 CHD patients received statin treatment (atorvastatin calcium 20 or 40 mg/day, rosuvastatin calcium 10 mg/day, pravastatin sodium 40 mg/day, or simvastatin 40 mg/day) regardless of whether a stent was implanted or not. Sixty-three CHD patients were followed after 3 months of regular statin treatment (detailed information about the statin treatment was listed in Additional files 3 and 4); 57 CHD patients refused to return for blood drawing. Considered the effect of Diabetes mellitus (DM) on lipids metabolism, the DM history of each patient was recorded. DM was diagnosed based on the National Cholesterol Education Program Adults Treatment Panel III or personal DM history. A total of 10 CHD patients with DM were followed in this study, including 5 patients used acarbose (2 of them also used glyburide or glipizide), 2 patients used insulin, and 3 patients used metformin, Chinese medicine and dietetic treatment respectively. The exclusion criteria included patients with any kinds of tumors and infections, severe liver or kidney diseases, uncontrolled hypertension, and severe heart failure.

### Plasma preparation

Venous blood was collected from each patient after overnight fasting. Fresh plasma was obtained by centrifuging at 3000 r/min at 4 °C for 10 min immediately, and then aliquoting the plasma for lipids measurement or storage at −80 °C for HDL isolation and apoCIII measurement.

### HDL isolation

As described previously [21], a HDL (d = 1.063-1.240 g/ml) sample of each patient was isolated from plasma by a sequential ultracentrifugation method. Briefly, plasma was adjusted to 1.240 g/ml by potassium bromide powder according to the Radding-Steining formula. The adjusted plasma samples were laid on the bottom of a centrifuge tube through KBr solution (d = 1.063 g/ml) and was centrifuged by 80 Ti Rotor ultracentrifuge (Beckman Coulter, USA) at the condition of 65000 r/min at 10 °C for 5 h. Finally the HDL sample was collected, desalted, freeze-dried, and resolved by deionized water for measurement.

### Lipids measurement

Lipid markers including TC, TG, HDL-c, LDL-c, apolipoprotein AI (ApoAI), apolipoprotein B (ApoB) and high sensitivity C-reactive protein (HsCRP) were measured by a biochemistry analyzer in the clinical laboratory centre of Fuwai hospital, as described previously [21]. And the plasma apoCIII concentrations and HDL-apoCIII were measured by using an ELISA kit (Abcam, UK). The concentrations of HDL samples were quantified by using BCA protein assay (Applygen Technologies Inc, China). The effect of the differences of HDL sample concentrations was considered, and all the ELISA results (ng/ml) of HDL-apoCIII were adjusted by the concentrations (ug/ml) of HDL samples, and the final HDL-apoCIII unit was reported in ug/mgHDL.

### Statistical analysis

All the data were expressed as mean ± standard deviation (SD) or percent (%). Normality was checked before any analysis. The differences between groups were analyzed by student *t*-test, paired *t*-test or Mann–Whitney *U* test, or Wilcoxon rank test, as appropriate. A multivariate logistic regression model was used to analyze the odds ratios and 95 % confidence interval of lipid markers for predicting CHD. The correlations between lipid markers were analyzed by Pearson or Spearman correlation. The statistical analysis was performed using SPSS version 17.0 (SPSS Inc., Chicago, Illinois). A *p*-value of <0.05 was considered to be statistically significant.

## Results

As shown Table 1, the elder males had a higher ratio of CHD (*p* < 0.05), and the results consist to epidemiologic features. Data stratified by sex were listed in Additional file 1. Totally, compared with non-CHD patients, CHD patients had significantly higher apoB and HsCRP, and significantly lower HDL-c and apoAI than non-CHD patients (*p* < 0.05). There were no significant differences of TC, TG and LDL-c between CHD and non-CHD groups in this study (*p* > 0.05). However, there was no significant difference of total plasma apoCIII between two groups (*p* > 0.05), but HDL-apoCIII was significantly higher in CHD patients than non-CHD patients (*p* < 0.01).

**Table 1:** Baseline characteristics of all the patients

Variables	Non-CHD	CHD	*p*
	(*n* = 80)	(*n* = 120)	
Male (%)	55.0	76.7	0.002
Age (years)	51.34 ± 8.35	55.52 ± 10.04	0.002
BMI (kg/m^2^)	26.19 ± 3.23	26.31 ± 3.50	0.697
Hypertension (%)	47.5	58.3	0.191
Diabetes (%)	11.3	19.2	0.247
TC (mmol/L)	4.71 ± 0.80	4.49 ± 0.94	0.088
TG (mmol/L)	1.62 ± 1.00	1.76 ± 0.84	0.079
HDL-c (mmol/L)	1.24 ± 0.36	1.02 ± 0.23	<0.001
LDL-c (mmol/L)	3.03 ± 0.78	2.88 ± 0.82	0.199
ApoAI (mmol/L)	1.48 ± 0.29	1.34 ± 0.24	0.001
ApoB (mmol/L)	1.07 ± 0.22	1.16 ± 0.31	0.020
Glucose(mmol/L)	5.64 ± 1.36	5.43 ± 1.56	0.327
HsCRP (mg/L)	1.75 ± 1.72	3.66 ± 3.58	<0.001
ApoCIII (mg/L)	12.68 ± 3.74	11.97 ± 4.94	0.275
HDL-apoCIII (ug/mgHDL)	21.00 ± 11.33	25.05 ± 12.98	0.024

Data are expressed as mean ± standard deviation or percent (%). CHD = coronary heart disease; BMI = body mass index; TC = total cholesterol; TG = triglyceride; HDL-c = high density lipoprotein cholesterol; LDL-c = low density lipoprotein cholesterol; Apo = apolipoprotein; HsCRP = high sensitivity C-reactive protein; HDL-apoCIII = apoCIII content in HDL

In order to identify the relationship between HDL-apoCIII and CHD, we analyzed the associations between various lipid markers and CHD by multivariate logistic regression analysis (Table 2). HDL-apoCIII was still an independent predictor of CHD after adjusting for other facors (*p* < 0.05). Besides, sex, age, HDL-c, apoB and HsCRP were also significant predictors of CHD (*p* < 0.05). According to the data, HDL-c has a negative, and HDL-apoCIII has a positive association with CHD.

**Table 2:** Odds ratios (OR) and 95 % confidence intervals (CI) of variables for predicting CHD

Variables	OR	95 % CI	*p*
Sex	3.02	1.20-7.59	0.019
Age	1.07	1.02-1.12	0.003
TC	1.03	0.33-3.22	0.961
TG	0.83	0.45-1.52	0.543
HDL-c	0.06	0.01-0.95	0.046
LDL-c	0.44	0.13-1.50	0.191
ApoAI	2.21	0.19-25.49	0.526
ApoB	9.48	1.01-88.75	0.049
ApoCIII	0.96	0.87-1.05	0.345
HDL-apoCIII	1.04	1.00-1.08	0.039
HsCRP	1.22	1.02-1.45	0.028

CHD = coronary heart disease; TC = total cholesterol; TG = triglyceride; HDL-c = high density lipoprotein cholesterol; LDL-c = low density lipoprotein cholesterol; Apo = apolipoprotein; HDL-apoCIII = apoCIII content in HDL

The following variables were entered in the multivariable regression model: sex, age, BMI, TC, TG, HDL-c, LDL-c, lipoprotein a, apoAI, apoB, HsCRP, apoCIII, HDL-apoCIII and concomitant disease hypertension and diabetes mellitus

Sixty-three patients were followed in the study after 3 months of regular statin treatment, and the baseline characteristics of them were consistent with the CHD patients without follow-up data (Additional file 2, *p* > 0.05). The plasma apoCIII, especially HDL-apoCIII, had a significant elevation after statin treatment (*p* < 0.05), whereas HDL-c and ApoAI were elevated, and TC, LDL-c and apoB were decreased significantly (Table 3, *p* < 0.001). Although a similar effect on lipids changes was found in different statins, rosuvastatin showed a much stronger effect on apoCIII markers than the others (Additional file 3). We did not find an obvious dose-dependent effect in different doses of atorvastain treatment (Additional file 4). As an inflammation factor, HsCRP (3.25 ± 3.38 mg/L vs 2.22 ± 2.49 mg/L) was also significantly decreased after statin treatment (*p* < 0.01). In further analysis, the effect of statin treatment on apoCIII markers were minor in CHD patients with DM (*p* > 0.05), but the effect on plasma apoCIII (11.14 ± 3.72 mg/L vs 12.95 ± 5.75 mg/L) and HDL-apoCIII (25.19 ± 15.39 ug/mgHDL vs 30.84 ± 16.92 ug/mgHDL) were significant in CHD patients without DM (Additional file 5, *p* < 0.05).

**Table 3:** Effect of statin treatment on lipid variables in CHD patients

Variables	Statin treatment (n = 63)		*p*
	Pre-treatment	Post-treatment	
TC (mmol/L)	4.45 ± 0.98	3.85 ± 0.87	<0.001
TG (mmol/L)	1.68 ± 0.77	1.54 ± 0.76	0.138
HDL-c (mmol/L)	1.05 ± 0.25	1.21 ± 0.54	<0.001
LDL-c (mmol/L)	2.84 ± 0.84	2.19 ± 0.69	<0.001
ApoAI(mmol/L)	1.37 ± 0.24	1.49 ± 0.30	<0.001
ApoB (mmol/L)	1.16 ± 0.34	0.89 ± 0.29	<0.001
ApoCIII (mg/L)	11.30 ± 4.10	12.93 ± 5.71	0.045
HDL-apoCIII (ug/mgHDL)	24.26 ± 14.80	29.35 ± 16.46	0.003

CHD = coronary heart disease; TC = total cholesterol; TG = triglyceride; HDL-c = high density lipoprotein cholesterol; LDL-c = low density lipoprotein; Apo = apolipoprotein; HDL-apoCIII = apoCIII content in HDL

**Fig. 1:**
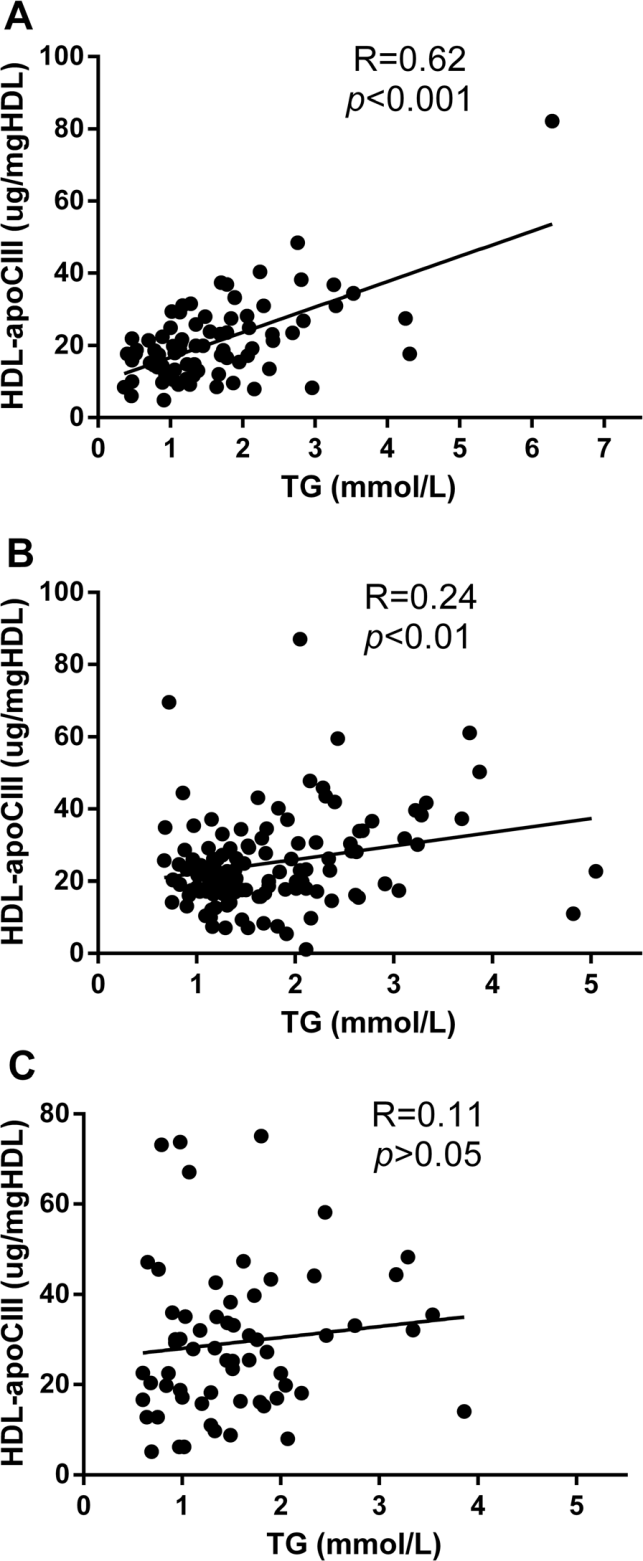
Correlations between HDL-apoCIII and plasma triglyceride in non-CHD patients (**a**) and CHD patients before (**b**) and after (**c**) statin treatment. HDL-apoCIII = apoCIII content in HDL; TG = triglyceride

HDL-apoCIII had significant correlations with plasma TG in non-CHD patients and CHD patients (Fig. 1a and b, *p* < 0.05), but this correlation disappeared after statin treatment (Fig. 1c, *p* > 0.05). However, plasma apoCIII had a moderate correlation with TG in non-CHD, CHD and statin treated CHD patients (r = 0.50, 0.59 and 0.58 respectively, *p* < 0.001); and HDL-apoCIII significantly correlated to plasma apoCIII regardless of whether statin drugs were used or not (r = 0.35, 0.31 and 0.35 respectively, *p* < 0.05). In this study, we did not find significant correlations between HDL-apoCIII or plasma apoCIII and HDL-c or LDL-c (*p* > 0.05).

## Discussion

In this study, we found that CHD patients had a significant higher level of HDL-apoCIII than non-CHD patients, and the association between HDL-apoCIII and CHD was still significant after adjusting for other variable factors. However, although conventional atherogenic lipid and inflammation factors were significantly decreased, there was a further elevation of HDL-apoCIII in CHD patients after statin treatment.

As an atherogenic factor, apoCIII exists in both HDL and apoB-lipoproteins, and transfers between them during the process of lipolysis [22–24]. Studies have found that apoCIII may participate in the initiation and progression of CHD through disturbing lipids metabolism, promoting the adhesion of monocyte to endothelial cell, and impairing the anti-apoptotic function of HDL [10–12, 15]. There were also some clinical studies found that HDL-apoCIII was significantly higher in CHD patients than in non-CHD patients, which can be used to predict the occurrence and progression of CHD, as well as the recurrent cardiovascular event [8, 16, 17]. In this study, in order to avoid potential effects of the concentration of HDL samples, we adjusted HDL-apoCIII values by the concentration of HDL after measuring apoCIII of HDL samples. And we also found a significantly higher HDL-apoCIII in CHD patients than non-CHD patients, and it still associated with CHD significantly after adjusting for other variable factors. To some extent, HDL-apoCIII was a better predictor of CHD than most conventional lipid markers.

This is the first time we quantitatively measured a significant elevation of HDL-apoCIII in CHD patients with adjustment of the effect of HDL sample, and established the association between HDL-apoCIII and CHD without the effect of lipid-lowering drugs. We also found an inverse association of HDL-c and HDL-apoCIII with CHD, a higher HDL-c predicts a lower risk of CHD, but a higher HDL-apoCIII predicts a higher risk of CHD, this finding was supported by the results of previous studies [4, 5, 17, 18]. Studies have found that HDL lost its anti-inflammatory and anti-atherogenic function in end-stage renal diseases or acute phase response [25–27]. Perhaps this condition also existed in CHD patients, and the elevated HDL-apoCIII may be partly responsible for the dysfunction of HDL in CHD patients, and the occurrence and progression of CHD. However, considered the inhibitory effect of apoCIII on lipolysis [24], the elevated HDL-apoCIII may delay the further lipolysis of HDL in CHD patients also. So, the biological function of HDL-apoCIII in CHD patients may need further more study.

Lipid-lowering drugs, especially statins, are famous for their cardiovascular protective role by decreasing atherogenic, elevating antiatherogenic lipids and inhibiting inflammation response [19, 20, 28, 29]. An earlier study indicated that lipid-lowering drugs (colestipol hydrochloride and niacin) significantly decreased conventional lipids and delayed the progression of CHD, but they significantly increased HDL-apoCIII [18]. Another study also found although most CHD patients received statin treatment, they still had higher apoCIII contents in plasma and HDL, and the elevated apoCIII in HDL was responsible for the impaired antiapoptotic effect of HDL [15]. There was little studies reported that atorvastatin can decrease plasma apoCIII [30], but this result was still controversial [31]. In this study, we found a similar effect of statin treatment on atherogenic lipids and inflammation factor as previous studies [19, 20, 29, 32], however, not as expected, plasma apoCIII, especially HDL-apoCIII has a significant elevation at the same time. The result was partly consistent to previous studies [15, 18], and all the results favored the theory of apoCIII transfer, increased lipolysis of TRL leads to increased transfer of apoCIII to HDL. However, no matter the elevation of HDL-apoCIII was naturally or caused by statin treatment, an excessive elevated HDL-apoCIII may impair, and even counteract the antiatherogenic effect of HDL and/or the benefits of statin treatment. All the results might explain why further elevating HDL-c could not increase heart benefits [6, 7], and why there were still residual cardiovascular risks after statin treatment in CHD patients [33, 34].

We found a similar effect of statin treatment on lipid markers in CHD patients with DM or not. Perhaps because of the small case number or the interaction of antidiabetic drugs, we did not find a significant effect of statin treatment on apoCIII markers in patients with DM (*n* = 10). Our results were inconsistent with a previous study, which found a decrease of apoCIII in plasma and HDL in DM patients after atorvastatin treatment [35]. DM patients have higher risk of CHD than non-diabetic patients [36, 37], although statin treatment reduces the risk of CHD by decreasing lipids, it also leads to an increased risk of DM [38]. To date, there is little information about the differences of effect of statins on lipid markers among CHD patients, DM patients and CHD patients with DM, maybe the effect are different among them.

As reported previously [39], we also found a significant correlation between HDL-apoCIII and TG. Perhaps because of the effect of dyslipidemia and statin drugs, the correlation was weaker in CHD patients, and disappeared after statin treatment. However, the correlation between apoCIII and TG or HDL-apoCIII was not affected by statin drugs. As a part of the theory of apoCIII transfer, we did not find a significant correlation between HDL-apoCIII and HDL-c or LDL-c.

### Limitations

An important limitation of our study was that there were no placebo controls because all the CHD patients received statin treatmen. However, to some extent, the self-control method and the consistency of the results to previous studies favor the accuracy of the study. Although ultracentrifuge is a golden standard to isolate HDL, and we adjusted potential effect of HDL samples on the apoCIII results as far as possible, there may be some bias existing inevitably during the procedure.

## Conclusions

Our study found that, HDL-apoCIII has a positive and significant association with CHD. Although statin treatment for CHD patients could decrease conventional atherogenic lipid markers significantly, HDL-apoCIII also has a significant elevation. The results may indicate a new aspect for studying CHD and evaluating the effect of statin treatment on the biological function of HDL in CHD patients.

## Additional files

Additional file 1:
**Baseline characteristics of all patients stratified by sex.** (DOC 39 kb) Additional file 2:
**Baseline characteristics of CHD patients with followed data or not.** (DOC 37 kb) Additional file 3:
**Effect of different types of statins on lipid variables in CHD patients.** (DOC 36 kb) Additional file 4:
**Effect of different doses of atorvastatin on lipid variables in CHD patients.** (DOC 36 kb) Additional file 5:
**Effect of statin treatment on lipid variables in CHD patients with DM or not.** (DOC 37 kb)

## Abbreviations

CHD: Coronary heart disease
DM: Diabetes mellitus
TC: Total cholesterol
TG: Triglyceride
HDL-c: High density lipoprotein cholesterol
LDL-c: Low density lipoprotein cholesterol
Apo: Apolipoprotein
HsCRP: High sensitivity C-reactive protein
HDL-apoCIII: apoCIII content in HDL
TRL: Triglyceride-rich lipoproteins
