# Supplementary material for: The association of HDL-apoCIII with coronary heart disease and the effect of statin treatment on it
Source: Lipids Health Dis. 2015 Oct 9;14:127. doi: 10.1186/s12944-015-0129-8 (PMC4600316; doi:10.1186/s12944-015-0129-8)
Supplement: Additional file 1: — Baseline characteristics of all patients stratified by sex. (DOC 39 kb) [file 12944_2015_129_MOESM1_ESM.doc]

**Additional file 1** Baseline characteristics of all patients stratified by sex

| Variables | Total (n=200) | | | Male (n=136) | | | Female (n=64) | | |
| --- | --- | --- | --- | --- | --- | --- | --- | --- | --- |
|  | Male  (n=136) | Female  (n=64) | *p* | Non-CHD  (n=44) | CHD  (n=92) | *p* | Non-CHD  (n=36) | CHD  (n=28) | *p* |
| Age (years) | 52.63±9.97 | 56.42±8.26 | 0.009 | 48.89±7.82 | 54.42±10.42 | 0.001 | 54.33±8.10* | 59.11±7.81* | 0.020 |
| BMI (kg/m^2^) | 26.59±3.57 | 25.57±2.86 | 0.032 | 26.82±3.45 | 26.47±3.64 | 0.589 | 25.40±2.79* | 25.78±2.97 | 0.600 |
| TC (mmol/L) | 4.43±0.83 | 4.90±0.94 | <0.001 | 4.36±0.79 | 4.58±0.88 | 0.141 | 4.87±0.67* | 4.94±1.22* | 0.802 |
| TG (mmol/L) | 1.78±0.92 | 1.53±0.86 | 0.063 | 1.70±0.80 | 1.95±1.12 | 0.148 | 1.21±0.65* | 1.93±0.94 | 0.001 |
| HDL-c (mmol/L) | 1.03±0.25 | 1.28±0.35 | <0.001 | 1.11±0.30 | 0.99±0.21 | 0.018 | 1.40±0.37* | 1.12±0.26* | 0.001 |
| LDL-c (mmol/L) | 2.85±0.74 | 3.13±0.89 | 0.019 | 2.79±0.68 | 2.96±0.85 | 0.204 | 3.10±0.68 | 3.17±1.12 | 0.799 |
| ApoAI (mmol/L) | 1.34±0.22 | 1.52±0.31 | <0.001 | 1.39±0.22 | 1.31±0.21 | 0.049 | 1.58±0.33* | 1.44±0.28* | 0.072 |
| ApoB (mmol/L) | 1.11±0.25 | 1.16±0.34 | 0.240 | 1.08±0.22 | 1.13±0.26 | 0.268 | 1.06±0.22 | 1.29±0.42 | 0.015 |
| HsCRP (mg/L) | 3.08±3.30 | 2.51±2.68 | 0.228 | 1.70±1.43 | 3.75±3.72 | <0.001 | 1.82±2.03 | 3.40±3.15 | 0.025 |
| ApoCIII (mg/L) | 11.92±4.65 | 12.98±4.11 | 0.119 | 11.33±4.85 | 13.14±3.98 | 0.034 | 12.13±3.40 | 14.07±4.71 | 0.072 |
| HDL-apoCIII  (ug/mgHDL) | 24.02±13.12 | 22.18±10.97 | 0.331 | 21.35±13.24 | 26.34±13.13 | 0.044 | 18.12±7.67* | 27.39±12.42 | 0.001 |

Data are expressed as mean ± standard deviation.

CHD = coronary heart disease; BMI = body mass index; TC = total cholesterol; TG = triglyceride; HDL-c = high density lipoprotein cholesterol; LDL-c = low density lipoprotein cholesterol; Apo = apolipoprotein; HsCRP = high sensitivity C-reactive protein; HDL-apoCIII = apoCIII content in HDL.

*Compared with males, *p*<0.05.
