# Supplementary material for: The association of HDL-apoCIII with coronary heart disease and the effect of statin treatment on it
Source: Lipids Health Dis. 2015 Oct 9;14:127. doi: 10.1186/s12944-015-0129-8 (PMC4600316; doi:10.1186/s12944-015-0129-8)
Supplement: Additional file 2: — Baseline characteristics of CHD patients with followed data or not. (DOC 37 kb) [file 12944_2015_129_MOESM2_ESM.doc]

**Additional file 2** Baseline characteristics of CHD patients with followed data or not

| Variables | Un-followed CHD (n=57) | Followed CHD (n=63) | *p* |
| --- | --- | --- | --- |
| Men (%) | 78.9 | 74.6 | 0.667 |
| Age (years) | 55.21±10.96 | 55.79±9.20 | 0.583 |
| Hypertension (%) | 57.9 | 58.7 | 1.000 |
| Diabetes (%) | 22.8 | 15.9 | 0.362 |
| TC (mmol/L) | 4.54±0.89 | 4.45±0.98 | 0.574 |
| TG (mmol/L) | 1.84±0.90 | 1.68±0.77 | 0.319 |
| HDL-c (mmol/L) | 0.99±0.21 | 1.05±0.25 | 0.179 |
| LDL-c (mmol/L) | 2.92±0.79 | 2.84±0.84 | 0.570 |
| ApoAI (mmol/L) | 1.31±0.23 | 1.37±0.24 | 0.169 |
| ApoB (mmol/L) | 1.17±0.27 | 1.16±0.34 | 0.930 |
| Glucose (mmol/L) | 5.61±1.89 | 5.27±1.17 | 0.668 |
| HsCRP (mg/L) | 4.14±3.76 | 3.25±3.39 | 0.240 |
| ApoCIII (mg/L) | 12.72±5.67 | 11.30±4.10 | 0.123 |
| HDL-apoCIII (ug/mgHDL) | 25.92±10.68 | 24.26±14.80 | 0.486 |

Data are expressed as mean ± standard deviation or percent (%).

CHD = coronary heart disease; BMI = body mass index; TC = total cholesterol; TG = triglyceride; HDL-c = high density lipoprotein cholesterol; LDL-c = low density lipoprotein cholesterol; Apo = apolipoprotein; HsCRP = high sensitivity C-reactive protein; HDL-apoCIII = apoCIII content in HDL.
