# Supplementary material for: The association of HDL-apoCIII with coronary heart disease and the effect of statin treatment on it
Source: Lipids Health Dis. 2015 Oct 9;14:127. doi: 10.1186/s12944-015-0129-8 (PMC4600316; doi:10.1186/s12944-015-0129-8)
Supplement: Additional file 3: — Effect of different types of statins on lipid variables in CHD patients. (DOC 36 kb) [file 12944_2015_129_MOESM3_ESM.doc]

**Additional file 3** Effect of different types of statins on lipid variables in CHD patients

| Variables | Atorvastatin (n=44) | | | Rosuvastatin (n=11) | | | Pravastatin (n=5) | | | Simvastatin (n=3) | | |
| --- | --- | --- | --- | --- | --- | --- | --- | --- | --- | --- | --- | --- |
|  | Pre-therapy | Post-therapy | *p* | Pre-therapy | Post-therapy | *p* | Pre-therapy | Post-therapy | *p* | Pre-therapy | Post-therapy | *p* |
| TC (mmol/L) | 4.38±0.85 | 3.70±0.89 | <0.001 | 4.83±1.37 | 4.34±0.72 | 0.135 | 4.13±1.03 | 4.22±0.84 | 0.785 | 4.49±1.27 | 3.55±0.24 | 0.292 |
| TG (mmol/L) | 1.70±0.80 | 1.55±0.73 | 0.203 | 1.61±0.82 | 1.23±0.43 | 0.103 | 1.81±0.70 | 1.89±0.88 | 0.670 | 1.52±0.60 | 1.96±1.68 | 0.554 |
| HDL-c (mmol/L) | 1.04±0.26 | 1.20±0.63 | 0.071 | 1.11±0.21 | 1.27±0.20 | 0.026 | 0.88±0.13 | 1.11±0.26 | 0.027 | 1.22±0.34 | 1.24±0.28 | 0.883 |
| LDL-c (mmol/L) | 2.78±0.71 | 2.07±0.64 | <0.001 | 3.18±1.19 | 2.59±0.76 | 0.046 | 2.62±0.86 | 2.56±0.78 | 0.836 | 2.76±1.18 | 1.77±0.30 | 0.203 |
| ApoAI(mmol/L) | 1.35±0.23 | 1.44±0.31 | 0.024 | 1.41±0.25 | 1.62±0.23 | 0.045 | 1.28±0.15 | 1.54±0.26 | 0.021 | 1.58±0.40 | 1.66±0.23 | 0.589 |
| ApoB (mmol/L) | 1.14±0.29 | 0.89±0.30 | <0.001 | 1.27±0.50 | 0.92±0.30 | 0.016 | 1.22±0.25 | 0.89±0.25 | 0.021 | 1.03±0.54 | 0.73±0.15 | 0.520 |
| ApoCIII (mg/L) | 11.06±3.72 | 12.69±5.97 | 0.116 | 11.38±3.54 | 13.18±4.85 | 0.148 | 11.52±3.91 | 14.99±7.17 | 0.279 | 14.17±10.80 | 12.16±3.05 | 0.745 |
| HDL-apoCIII  (ug/mgHDL) | 25.44±16.51 | 29.21±18.10 | 0.094 | 22.74±11.12 | 31.20±12.81 | 0.004 | 21.05±7.61 | 30.18±13.13 | 0.179 | 17.78±8.05 | 23.13±10.77 | 0.337 |

Data are expressed as mean ± standard deviation.

CHD = coronary heart disease; TC = total cholesterol; TG = triglyceride; HDL-c = high density lipoprotein cholesterol; LDL-c = low density lipoprotein cholesterol; Apo = apolipoprotein; HDL-apoCIII = apoCIII content in HDL.

Notes: There are 4 patients used atorvastatin 40mg/d and 40 used 20mg/d in the atorvastatin group, 11 patients used rosuvastatin 10mg/d in the rosuvastatin group, 5 patients used pravastatin 40mg/d in the pravastatin group and 3 patients used simvastatin 40mg/d in the simvastatin group.
