# Supplementary material for: The association of HDL-apoCIII with coronary heart disease and the effect of statin treatment on it
Source: Lipids Health Dis. 2015 Oct 9;14:127. doi: 10.1186/s12944-015-0129-8 (PMC4600316; doi:10.1186/s12944-015-0129-8)
Supplement: Additional file 4: — Effect of different doses of atorvastatin on lipid variables in CHD patients. (DOC 36 kb) [file 12944_2015_129_MOESM4_ESM.doc]

**Additional file 4** Effect of different doses of atorvastatin on lipid variables in CHD patients

| Variables | Atorvastatin 20mg/d (n=40) | | | Atorvastatin 40mg/d (n=4) | | |
| --- | --- | --- | --- | --- | --- | --- |
|  | Pre-therapy | Post-therapy | *p* | Pre-therapy | Post-therapy | *p* |
| TC (mmol/L) | 4.36±0.87 | 3.71±0.91 | 0.001 | 4.61±0.81 | 3.64±0.76 | 0.300 |
| TG (mmol/L) | 1.68±0.83 | 1.58±0.76 | 0.377 | 1.83±0.50 | 1.32±0.35 | 0.051 |
| HDL-c (mmol/L) | 1.05±0.26 | 1.21±0.66 | 0.092 | 0.96±0.21 | 1.08±0.26 | 0.035 |
| LDL-c (mmol/L) | 2.75±0.72 | 2.06±0.66 | <0.001 | 3.05±0.73 | 2.19±0.43 | 0.204 |
| ApoAI(mmol/L) | 1.37±0.23 | 1.47±0.31 | 0.018 | 1.20±0.24 | 1.19±0.24 | 0.942 |
| ApoB (mmol/L) | 1.13±0.29 | 0.89±0.31 | <0.001 | 1.21±0.31 | 0.89±0.15 | 0.260 |
| ApoCIII (mg/L) | 11.28±3.76 | 12.75±6.13 | 0.185 | 8.90±2.82 | 12.08±4.72 | 0.308 |
| HDL-apoCIII  (ug/mgHDL) | 26.80±16.48 | 30.53±18.21 | 0.129 | 11.90±10.43 | 16.01±11.43 | 0.246 |

Data are expressed as mean ± standard deviation.

CHD = coronary heart disease; TC = total cholesterol; TG = triglyceride; HDL-c = high density lipoprotein cholesterol; LDL-c = low density lipoprotein cholesterol; Apo = apolipoprotein; HDL-apoCIII = apoCIII content in HDL.

Note: There were no significant differences of lipid markers between 20mg atorvastatin group and 40mg atorvastatin group before or after treatment.
