# Supplementary material for: The association of HDL-apoCIII with coronary heart disease and the effect of statin treatment on it
Source: Lipids Health Dis. 2015 Oct 9;14:127. doi: 10.1186/s12944-015-0129-8 (PMC4600316; doi:10.1186/s12944-015-0129-8)
Supplement: Additional file 5: — Effect of statin treatment on lipid variables in CHD patients with DM or not. (DOC 37 kb) [file 12944_2015_129_MOESM5_ESM.doc]

**Additional file 5** Effect of statin treatment on lipid variables in CHD patients with DM or not

| Variables | CHD with DM (n=10) | | | CHD without DM (n=53) | | |
| --- | --- | --- | --- | --- | --- | --- |
|  | Pre-therapy | Post-therapy | *p* | Pre-therapy | Post-therapy | *p* |
| TC (mmol/L) | 3.92±0.67 | 3.51±0.62 | 0.198 | 4.54±1.01 | 3.92±0.89 | <0.001 |
| TG (mmol/L) | 2.08±1.10 | 1.70±0.91 | 0.303 | 1.61±0.68 | 1.51±0.73 | 0.297 |
| HDL-c (mmol/L) | 0.90±0.24 | 0.99±0.27 | 0.050 | 1.07±0.24 | 1.25±0.57 | <0.001 |
| LDL-c (mmol/L) | 2.35±0.53 | 1.98±0.49 | 0.075 | 2.93±0.86 | 2.23±0.72 | <0.001 |
| ApoAI(mmol/L) | 1.23±0.23 | 1.39±0.30 | 0.036 | 1.39±0.23 | 1.51±0.30 | 0.003 |
| ApoB (mmol/L) | 1.05±0.35 | 0.92±0.22 | 0.344 | 1.18±0.33 | 0.88±0.30 | <0.001 |
| ApoCIII (mg/L) | 12.17±5.89 | 12.81±5.77 | 0.765 | 11.14±3.72 | 12.95±5.75 | 0.041 |
| HDL-apoCIII  (ug/mgHDL) | 19.31±10.43 | 21.44±11.47 | 0.555 | 25.19±15.39 | 30.84±16.92 | 0.004 |

Data are expressed as mean ± standard deviation.

CHD = coronary heart disease; DM = diabetes metabolism; TC = total cholesterol; TG = triglyceride; HDL-c = high density lipoprotein cholesterol; LDL-c = low density lipoprotein; Apo = apolipoprotein; HDL-apoCIII = apoCIII content in HDL.

Note: Of 10 CHD patients with DM were followed in this study, 5 patients used acarbose (2 of them also used glyburide or glipizide), 2 patients used insulin, and 3 patients used metformin, Chinese medicine and dietetic treatment respectively.
